# Delirium, Antipsychotics and Death in the time of COVID-19

**DOI:** 10.1192/j.eurpsy.2023.863

**Published:** 2023-07-19

**Authors:** R. Softic, N. Becarevic

**Affiliations:** of psychiatry, University Clinical Center Tuzla, Tuzla, Bosnia and Herzegovina

## Abstract

**Introduction:**

Delirium is an acute, transient, global organic disorder of CNS functioning resulting in impaired consciousness, attention, and other cognitive functions. Causes of delirium are multifactorial and can be unrecognized in 2/3 of cases. It is recommended to use as few psychotropic medications as possible because many of them can worsen delirium. Antipsychotics are not recommended as a drug of first choice.

**Objectives:**

To present rate of delirium regarding to treatment and outcome.

**Methods:**

A retrospective observational study was conducted in the department of consultative psychiatry of the University Clinical Center Tuzla during the one-year period of the COVID-19 pandemic.

**Results:**

761 calls from different clinics of the University Clinical Center Tuzla were received in one year period. Delirium was diagnosed in 213 patients (28%). The total number of deaths was 147 (19.3%), the number of deaths in patients with delirium was 88 (41.3%). Antipsychotics were used in 137 (64%) patients with delirium. Death as an outcome was more common in patients treated with antipsychotics (64%) p˂ 0.05. The most used antipsychotic was Promazine 94 (44.1%). Number of deaths in patients with delirium treated with Promazine was 42 (44.7%) p ˂ 0.05.

**Conclusions:**

In patients with delirium mortality is significantly higher in those treated with antipsychotics, especially when treated with Promazine. The choice of antipsychotic medications should be made according to pharmacological properties and the clinical context.

**Disclosure of Interest:**

None Declared

